# ECMO for Cardiac Rescue after Accidental Intravenous Mepivacaine Application

**DOI:** 10.1155/2012/491692

**Published:** 2012-08-27

**Authors:** Michael Froehle, Nikolaus A. Haas, Guenther Kirchner, Deniz Kececioglu, Eugen Sandica

**Affiliations:** ^1^Zentrum angeborener Herzfehler, Herz und Diabetes Zentrum, North-Rhine Westphalia, Georgstrasse 11, 32545 Bad Oeynhausen, Germany; ^2^Department für Chirurgie angeborener Herzfehler, Herz und Diabetes Zentrum, North-Rhine Westphalia, Georgstrasse 11, 32545 Bad Oeynhausen, Germany

## Abstract

Mepivacaine is a potent local anaesthetic and used for infiltration and regional anaesthesia in adults and pediatric patients. Intoxications with mepivacaine affect mainly the CNS and the cardiovascular system. We present a case of accidental intravenous mepivacaine application and intoxication of an infant resulting in seizure, broad complex bradyarrhythmia, arterial hypotension and finally cardiac arrest. The patient could be rescued by prolonged resuscitations and a rapid initiation of ECMO and survived without neurological damage. The management strategies of this rare complication including promising other treatment options with lipid emulsions are discussed.

## 1. Introduction

Mepivacaine is a very potent local anaesthetic and used in many infiltration and regional anaesthesia indications [1]. Systemic toxic reactions affect mainly the CNS and the cardiovascular system [2, 3]. Initial symptoms of toxic reactions are usually dizziness, paresthesia in the mouth, numbness of the tongue, excessive hearing acuity, tinnitus, and visual disturbances as well as speech disturbances. Muscle twitching or tremors are more serious and precede unconsciousness and generalized grand mal convulsions, which usually last a few seconds to few minutes [4, 5]. Hypoxia and excessively high arterial carbon dioxide may result, explained by increased muscle activity with impaired respiration. In severe cases, a respiratory failure may occur. Acidosis, hyperkalemia, hypocalcaemia, and hypoxia enhance the toxic effects. The signs of toxic symptoms in the central nervous system generally precede the toxic cardiovascular effects [3, 5, 6]. The toxic cardiac effects follow a biphasic pathway. At lower concentrations, sympathetic nervous system activation during the CNS excitatory phase can lead to hypertension and tachycardia. This may conceal the direct myocardial depressant effects at higher concentrations followed by ventricular arrhythmias, myocardial conduction delays, and profound contractile dysfunction ultimately leading to cardiovascular collapse necessitating extended periods of resuscitation [2, 5]. We present a case of an accidental intravenous mepivacaine application in an infant resulting in generalized convulsion and bradyarrhythmia followed by profound, broad complex bradyarrhythmia, and ultimately cardiac arrest who could be rescued by a long period of resuscitation and extracorporeal membraneoxygenation (ECMO).

## 2. Case Report

**Figure 1:**
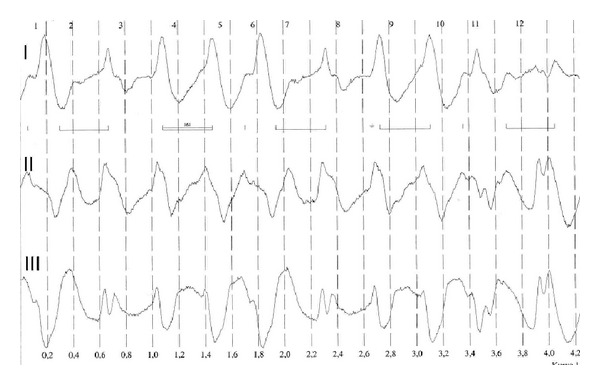
Broad complex bradyarrhythmia.

The nine-month-old female patient (6.9 kg, 74 cm) presented with a double outlet right ventricle (DORV) with atresia of the mitral valve and univentricular physiology. A pulmonary artery banding and atrioseptectomy was performed elsewhere for palliation at the age of 1 month. For further palliation, a bidirectional cavopulmonary anastomosis was performed. After successful surgical treatment and extubation, the patient showed moderate cyanosis with transcutaneous saturation below 80% despite additional oxygen (1-2 L/min). Therefore, a cardiac catheterization was performed under sedation with ketamine and propofol infusion to evaluate the surgical anastomosis and rule out treatable residual defects. Due to a preparation error, the syringes for contrast media were filled with mepivacaine 2%. During the test-dose application, a total dose of 6 mL (6 mLs mepivacaine 2% (120 mg = 20 mg/kg body weight) was accidentally injected into the central venous catheter in the right internal jugular vein. Immediately a generalized tonic-clonic seizure occurred that stopped spontaneously within a minute and after phenobarbitone administration (10 mg/kg). In addition, the electrocardiogram showed a broad complex bradyarrhythmia (see Figure 1) and severe arterial hypotension leading ultimately to asystole. Cardiopulmonary resuscitation was initiated immediately using repetitive and increasing epinephrine boluses (1 to 5 mLs adrenaline 1 : 10000, total of 22 doses over 40 minutes), as well as a continuous adrenaline infusion at increasing rate (up to 2 *μ*g/kg/min) to stabilize the blood pressure. Additionally, 10% calcium (12 mLs), 5.55% calcium chloride (11 mLs), magnesium 10% (14 mLs), and 4.2% sodium bicarbonate (120 mLs) as well as amiodarone 5 mg/kg were administered according to arterial blood gas analysis and with the idea to neutralize the effects of the local anaesthetic. These measures resulted in a brief period of tolerable blood pressures and adequate heart rate with broad QRS complexes. Transcutaneous saturations of about 85% were achieved during initially mask ventilation and after endotracheal intubation. However, despite continuous high-dose epinephrine application and additional noradrenaline infusion (up to 2 *μ*g/kg/min), it was not possible to maintain adequate and persistent blood pressure. Therefore, the decision was made to establish the implantation of a venoarterial extracorporeal membrane oxygenation (ECMO). Cannulation was performed about 90 minutes after the mepivacaine injection via the internal jugular vein. Due to the cardiac anatomy and after cardiac surgery, an transthoracic approach was chosen. We used a Biomedics 10 Fr canula for arterial access and a Pediatric VR 20 FR 1/4 canula for venous cannulation. Flow was generated by a CentriMag pump and 1/4 heparin-coated tubing, and the oxygenator used was a Hilite 2800 Paed. Priming was performed with normal saline, 20% of human albumin, and packed blood cells to achieve a haematocrit of about 30%. The initial flow used was 156 mLs/kg/min and adjusted to serum lactate levels, blood pressure, and clinical assessment. The small child improved significantly within the following hours, and ECMO flow could be reduced to 30% after 24 hrs. There were no significant changes in electrolytes, and renal function was adequate under moderate diuretic stimulation. After the initiation of the ECMO flow, the catecholamine support could be weaned rapidly. The clinical course was complicated by complete left-sided atelectasis and respiratory compromise. Therefore the ECMO was explanted with some delay after 4 days and the patient could be extubated one day after decannulation. The subsequent postoperative course was uneventful, and the patient was discharged home 14 days after the event in an excellent general condition without neurological abnormalities.

## 3. Discussion

Mepivacaine is a well-established widely used local anesthetic of the anilide type with reasonably rapid onset and medium duration of action [1]. In general, local anesthetics work through reversible binding at sodium channels, and signs and symptoms of toxicity depending of the drug concentration include central nervous system and cardiovascular effects [6]. The side effects at high doses or after intravascular injection in literature are well described [2–6, 9] and include nausea and vomiting, arterial hypotension, paresthesias, vertigo, convulsions, suppression of the function of CNC, neuropathy, arachnoiditis, bradycardia, cardiac arrest, cardiac arrhythmias, respiratory depression, and many more [2, 5]. The critical threshold dose is a concentration of 5-6 micrograms mepivacaine-hydrochloride per mL blood plasma. The lethal dose (LD50) in mouse studies is for intravenous application 35 mg/kg and for subcutaneous application 280 mg/kg. Mepivacaine side effects seem to be less in pediatric patients when correctly used and less compared to other local anesthetic agents [2, 5, 9].

Most available information on cardiac toxicity is from animal studies and case reports. The principal mechanism of cardiac toxicity relates to the blockade of myocardial voltage-dependent sodium channels, leading to an increase in the PR interval and QRS duration. This is followed by a dose-dependent prolongation of conduction time and eventual depression of spontaneous pacemaker activity. Persistent sodium channel blockade predisposes to reentrant arrhythmias. These electrophysiological effects are compounded by a direct negative inotropic effect of local anesthetic drugs [2]. The cardiac toxicity is extremely refractory to most conventional resuscitative techniques and drugs; therefore, extended periods of continuous resuscitation measures have to be applied to support the patients circulation when toxicity occurs. In selected cases, as in our patient, the institution of cardiopulmonary bypass was reported as the ultimate treatment option as a bridge to recovery. For many years, the institution of cardiopulmonary bypass was the first-line treatment [3].

Resuscitation ECMO is well established in many centres for various reasons including acute cardiac failure due to myocarditis, cardiomyopathy, postoperative failure, after resuscitation, and arrhythmias [10–13]. The first report suggesting ECMO for intoxications has been published in 1994 [14]. Other cases of successful ECMO for various intoxications with cardiotoxic drugs have been described [15–18]. In general, intact neurological survival can be achieved when ECMO initiation can be obtained without significant delay and according to standardised hospital protocols. Despite the tragic and inadvertent medication error, rapid recognition of the underlying cause, persistent resuscitation, and consequent ECMO initiation without significant delay resulted in an excellent outcome in our patient without any neurologic sequelae.

Nowadays another potential treatment option for serious side effects of mepivacaine intoxication is in discussion. Based on the literature, an intravenous therapy with high doses of lipid emulsions (IVLEs) seems to be a promising therapeutic approach. Due to the nature of the underlying problem, only single case publications are available as well as some animal studies; the data however seem to support this therapeutic approach with promising results [9, 18, 19]. The first case involved a 58-year-old male who developed seizures and asystolic cardiac arrest after receiving regional anesthesia with 40 mLs solution containing 20 mLs mepivacaine 1.5% and 20 mLs bupivacaine 0.5%. After resuscitation, medication with propofol, epinephrine, atropine, amiodarone, vasopressin, and monophasic defibrillation follows subsequently IVLE 20% 100 mLs in conjunction with ongoing resuscitation efforts, with return of spontaneous circulation. The IVLE 20% was then administered at 0.5 mLs/kg/min for 2 hours. The patient recovered without resultant neurologic deficits or signs or symptoms of adverse effects of IVLE use when followed for 2 weeks [20]. Charbonneau et al. described the successful use of intravenous lipid in a mepivacaine intoxication at a 19-year-old patient with an axillary brachial plexus block. A local anaesthetic solution with 50 mLs of 2% mepivacaine was injected. A few minutes later, the patient complained of dysarthria followed by myoclonia of the upper limbs that progressively increased during the next minutes. The symptoms did not lead to any clinical improvement and immediately disappeared after intravenous administration of 100 mLs Medialipide 20%. After injection, the clinical examination was normal and it was decided to proceed with surgery which was uneventful [18]. Another case report of treatment with intravenous lipid emulsions after intoxication with local anaesthetic (bupivacaine) in an infant (40 days old, 4.96 kg weight) was published by Shah et al. in 2009. After a caudal epidural block with a first test dose of 0.5 mls bupivacaine 0.25% and epinephrine 1 : 200.000 wasinjected in 2-3 minutes, 3.5 mLs bupivacaine 0.25% and epinephrine in incremental doses. Immediately, the heart rate increased, and the ECG, the ST-Segment elevation, and T-wave inversion occurred. The end tidal carbon dioxide concentration decreased to 20 mmHg, the oxygen saturation decreased to 80%, and the blood pressure dropped to 31/19 mmHg. The inhalational anaesthetic was turned off, and the patient was ventilated with 100% oxygen. Epinephrin, and albumin 5% were given, and the saturation improved to 100%, but the ECG changes persisted. So 10 mLs of 20% lipid emulsions was given over 1-2 minutes approximately 5 minutes after the bupivacaine injection. The hemodynamic parameters stabilized, and the ECG reverted back to baseline within a few minutes. The following treatment was uneventful, and the patient was dismissed without neurological or cardiac deficits a few days later [21]. Zausig et al. could show in an animal study that the lipid emulsion had no influence on the recovery time from local anaesthetics induced cardiac arrest to the return of first signs of cardiac activity. However, after the return of heart rhythm, lipid emulsions significantly decreased the recovery time for heart rate and RPP (RPP = (left ventricular systolic pressure − left ventricular diastolic pressure) × heart rate) in bupivacaine-induced cardiac toxicity, but not in mepivacaine- or ropivacaine-induced cardiac toxicity [22]. So in conclusion intravenous lipid emulsions may show an effect on neurological results of mepivacaine overdose, but their effect in the treatment of cardiac symptoms is not clear. A systemic review from Jamaty et al. 2010 shows only good results with mepivacaine in combination with other local anaesthetic agents (prilocaine 1%, bupivacaine 0.5%) for cardiac symptoms. They came to the conclusion that there was a benefit for intravenous lipid therapy, mainly after bupivacaine intoxication [23]. In summary the potential effect of intravenous lipid emulsion application for cardiac symptoms after mepivacaine overdose alone remains unclear.

**Figure 2:**
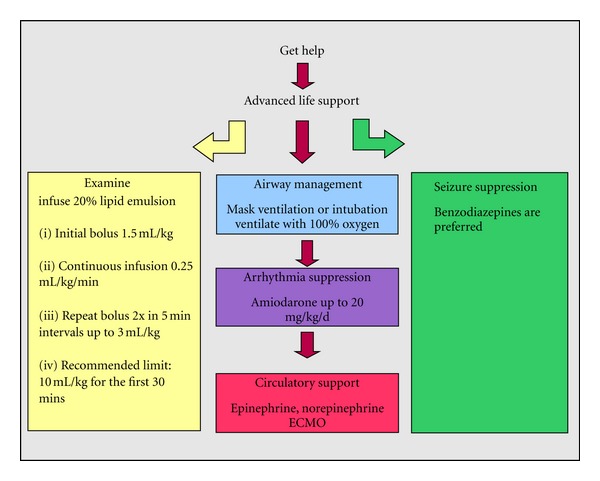
Treatment algorithm for side effects after intoxication with local anaesthetics modified after http://www.lipidrescue.org/ [7] and Weinberg [8].

The optimal dose of IVLE has not been established yet and the potential risks of administering relatively high doses are uncertain. Despite these uncertainties, it seems appropriate to administer lipid emulsion during advanced cardiac life support and after failure prior to cardiopulmonary bypass [19] as outlined by http://www.lipidrescue.org/ (Figure 2).

In our case, we did not use the intravenous lipid emulsions therapy, mainly because we are not aware of the potential benefits of this therapeutic option. In addition, our patient stabilized after ECMO implantation. Today, based on the literature published, we would certainly consider this treatment if a similar case would occur. To prevent recurrence, our preparation protocol for contrast application was modified accordingly.

## 4. Conclusion

This case illustrates that continuous supportive therapy of the cardiovascular system over an extended period of time is the key principle in comparable cases; very high cumulative doses of epinephrine and other catecholamines may be necessary to maintain adequate blood pressure and circulation. Even after prolonged resuscitation, ECMO can successfully maintain cardiac output and vital organ perfusion while allowing time for drug redistribution, metabolism, and clearance. In addition, therapy with lipid emulsions infusion for intoxication with local anesthetics may offer an additional and promising approach; however, experience and case reports in pediatric patients are missing.
